# Renal Infarct After Endovascular Abdominal Aortic Aneurysm Repair: Consider in Back Pain Differential

**DOI:** 10.5811/cpcem.2019.10.43623

**Published:** 2020-01-02

**Authors:** Sophia Y. Liu, Anthony Hackett

**Affiliations:** Carl R. Darnall Army Medical Center, Department of Emergency Medicine, Fort Hood, Texas

## Abstract

As hypertension, obesity, and hyperlipidemia become more widespread, the prevalence of abdominal aortic aneurysms (AAA) has also increased.^1^ Traditionally those with multiple comorbidities – also those with greatest AAA mortality – were considered too high risk for operative repair. In recent decades, however, endovascular abdominal aortic aneurysm repair (EVAR) has become a popular option, especially for high-risk patients. Overall, short-term outcomes are comparable to traditional open repair despite higher patient baseline risk. However, EVAR comes with its own risks, which the emergency physician should be aware of. Here, we present a rare complication of EVAR: device thrombosis with subsequent renal infarct.

## CASE PRESENTATION

**Image 1:**
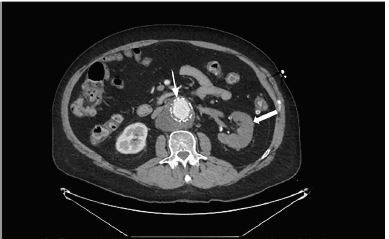
Computed tomography with contrast in axial view demonstrating thrombosed endoluminal aortic stent (thin arrow) and infarcted left kidney (thick arrow).

**Image 2:**
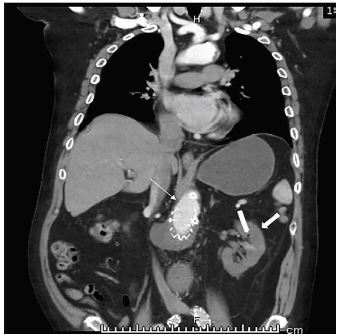
Computed tomography with contrast in coronal view demonstrating thrombosed endoluminal aortic stent (thin arrow) and infarcted left kidney (thick arrow) with intraparenchymal air (tab).

The image shows a left renal infarct two months after an endovascular abdominal aortic aneurysm repair (EVAR). A 50-year-old male with a history of lupus, chronic obstructive pulmonary disease, and coronary artery disease presented with four hours of acute back and groin pain. The patient had no history of coagulopathy or prior thromboembolism. His exam showed diffuse abdominal tenderness and was otherwise non-focal with good distal perfusion. Vitals were within normal limits and stable throughout evaluation. His laboratory values were normal except for a leukocytosis of 12×10^3^ per microliter (μL) (4.5–11.0×10^3^/μL) and a serum creatinine of 1.59 milligrams per deciliter (mg/dL) (0.6–1.3 mg/dL) with normal coagulation studies. Subsequent computed tomography angiogram of the aorta was performed, demonstrating a thrombosed endoluminal stent and a left kidney with intraparenchymal gas, indicating ischemic changes (Image 1 and 2).

## DISCUSSION

EVAR has become more common in recent decades as the technology has improved. This is an especially viable option for high-risk patients with comorbidities who may not tolerate open repair. Overall, EVAR has a lower 30-day mortality, perioperative morbidity, and a shorter recovery period compared to open repair. Despite higher-risk patients, endovascular and open repair have similar long-term outcomes and survival.^2^ Known complications of EVAR include the following: device migration; mechanical failures; graft infection; end-organ ischemia; and endoleaks (failure of the graft with continued expansion of the aneurysm from migration, graft porosity, aneurysmal dilation, collateral flow, or improper anchoring of the graft).

Bearing in mind these known complications, patients require lifelong surveillance.^3^ Renal infarction has been reported with endovascular repair, ranging from 2.5–6.4%, usually from stent occlusion of the accessory renal artery.^4^ Graft occlusion or restenosis is a relatively rare complication, with rates as low as <0.005%.^5^,^6^ As EVAR becomes more common and patients are surviving longer, emergency physicians should consider these potentially catastrophic complications during their workup of these patients presenting with severe back pain.

**CPC-EM Capsule**

What do we already know about this clinical entity?

Endovascular abdominal aortic aneurysm repair (EVAR) comes with known complications requiring life-long surveillance. Graft restenonsis however, is a very rare complication, occurring in <0.005%.

What is the major impact of the image(s)?

Renal infarct in EVAR is usually due to renal artery occlusion by the graft itself. Here, the infarct was secondary to graft thrombosis, highlighting another cause of morbidity and mortality.

How might this improve emergency medicine practice?

This image demonstrates a rare complication of an increasingly used intervention, thereby highlighting the need for caution by emergency physicians in this growing patient population.
